# Genetically predicted plasma metabolites mediate the causal relationship between gut microbiota and primary immune thrombocytopenia (ITP)

**DOI:** 10.3389/fmicb.2024.1447729

**Published:** 2024-10-28

**Authors:** Yang Hong, Cuilin Zhang, Kai Shen, Xiaoqing Dong, Bing Chen

**Affiliations:** Department of Hematology, Nanjing Drum Tower Hospital, Affiliated Hospital of Medical School, Nanjing University, Nanjing, China

**Keywords:** primary immune thrombocytopenia, plasma metabolites, gut microbiota, Mendelian randomization, mediation analyses

## Abstract

**Background:**

Primary immune thrombocytopenia (ITP) is an immune-mediated hematologic disorder characterized by a reduction in platelet count, increasing the risk of bleeding. Recent studies have indicated a close association between alterations in gut microbiota and the development of ITP. However, the mechanisms by which gut microbiota influence the occurrence and progression of ITP through plasma metabolites remain poorly understood. Evidence suggests extensive interactions between gut microbiota and plasma metabolites, implying a potential role for gut microbiota in influencing ITP through alterations in plasma metabolites, which requires further investigation.

**Methods:**

In this study, summarized GWAS data (including 211 gut microbiota taxa, 1,400 plasma metabolites or ratios, and an ITP patient cohort) were retrieved from the MiBioGen and GWAS Catalog databases. Using a two-sample Mendelian randomization (MR) approach, we screened gut microbiota and plasma metabolites potentially causally related to ITP. We further identified plasma metabolites serving as mediators through which gut microbiota affect ITP and calculated the strength of the mediation effect. To ensure result stability, we primarily used the inverse variance weighted (IVW) method as the main judgment index. We also utilized MR Egger and inverse variance weighted methods to detect heterogeneity in the results, and employed MR-Egger and MR-PRESSO methods to assess the presence of pleiotropy.

**Results:**

Though two-sample MR analysis, 8 gut microbiota taxa were found to have causal relationships with ITP. After excluding six plasma metabolites with pleiotropy, 39 plasma metabolites were found to be causally related to ITP (*P* < 0.05). Eleven plasma metabolites were identified as having causal relationships between gut microbiota and plasma metabolites. Finally, using the delta method, it was calculated that Sphingomyelin levels (8.0%, 95%CI: 0.9% to 11.5%, *P* = 0.047) and Glucose-to-mannose ratio (6.5%, 95%CI: 0.7% to 9.5%, *P* = 0.039) are intermediates for Intestinimonas influencing ITP, while Bilirubin (Z,Z) to etiocholanolone glucuronide ratio (5.6%, 95%CI: 4.7% to 6.9%, *P* = 0.043) is an intermediate for Senegalimassilia influencing ITP.

**Conclusion:**

Gut microbiota can influence the development of ITP through changes in plasma metabolites. Sphingomyelin levels, Glucose-to-mannose ratio, and Bilirubin (Z,Z) to etiocholanolone glucuronide ratio are newly discovered intermediates through which gut microbiota influence ITP, providing potential indicators and targets for clinical diagnosis and treatment. This study highlights the intricate relationship between gut microbiota and plasma metabolites in the context of ITP, suggesting new avenues for clinical diagnosis and treatment.

## Introduction

Primary immune thrombocytopenia (ITP) is an autoimmune disorder characterized by a reduced platelet count, leading to increased bleeding and bruising (Rodeghiero, 2023). The incidence of ITP is approximately 3.3 per 100,000 adults annually, with a slightly higher prevalence in females and individuals over 60 years of age (Bussel et al., 2023). The pathogenesis of ITP is primarily due to the immune system erroneously targeting and destroying platelets. Autoantibodies, particularly those against platelet glycoproteins such as GPIIb/IIIa and GPIb/IX, play a central role in this process (Semple et al., 2020). In addition to antibody-mediated destruction, T-cells and the accumulation of certain metabolites may directly or indirectly lyse platelets or impair megakaryocyte function, leading to reduced platelet production (Malik et al., 2023; Zhang et al., 2022). Known risk factors for ITP include viral infections, certain medications, and genetic predispositions, as indicated by familial cases and associations with specific HLA types (Cines, 2023).

Emerging research highlights the role of gut microbiota in the pathogenesis of ITP. Dysbiosis, or imbalance in the gut microbiota, may influence immune responses and contribute to the development and progression of ITP (Wang et al., 2021; Liu et al., 2020; Wang et al., 2023). However, the precise mechanisms underlying the influence of gut microbiota on ITP remain unclear and warrant further investigation.

Metabolites play a significant role in the pathogenesis of autoimmune diseases, including ITP (Zhang et al., 2022; Yang and Cong, 2021). Recent research has shown that plasma metabolic disturbances can influence immune cell function and contribute to the dysregulation seen in autoimmune conditions (Fernández-Ochoa et al., 2020). In autoimmune diseases, metabolites such as short-chain fatty acids (SCFAs), amino acids, and lipid mediators can modulate immune responses, either exacerbating or alleviating inflammation (Rasouli-Saravani et al., 2023; Mondanelli et al., 2019). In the context of ITP, studies have identified specific metabolites that may be involved in disease progression and severity (Wen et al., 2022). However, the precise roles of these metabolites in ITP remain elusive. The gut microbiota, a complex community of microorganisms residing in the gastrointestinal tract, significantly impacts the host's metabolic landscape through the production and modulation of various metabolites (Honda and Littman, 2016). For instance, gut microbiota ferments dietary fibers to produce SCFAs such as acetate, propionate, and butyrate, which play critical roles in maintaining gut barrier integrity, modulating immune responses, and providing energy sources for colonic cells. These SCFAs can enter the bloodstream, influencing systemic metabolic processes and exerting anti-inflammatory effects crucial in managing autoimmune diseases (Du et al., 2022). Additionally, gut bacteria modify primary bile acids into secondary bile acids, which regulate host metabolism and immune function by interacting with receptors like the farnesoid X receptor and G protein-coupled bile acid receptor 1 (Sepe et al., 2016). Gut microbiota also metabolizes tryptophan into various metabolites that modulate immune responses and intestinal health. In the context of lipid metabolism, gut bacteria influence sphingolipid levels, which are involved in cell signaling and immune regulation, and fatty acid profiles, impacting systemic inflammation and immune function (Rooks and Garrett, 2016). These findings suggest a complex interaction between microbial communities and host metabolism in the pathogenesis of autoimmune diseases. Therefore, it is reasonable to speculate that causal relationships may exist between gut microbiota, plasma metabolites, and ITP. Our study aims to elucidate these potential associations and identify specific metabolites that could serve as valuable tools for early diagnosis and potential clinical treatment.

Mendelian randomization (MR), an approach that utilizes genetic variants as instrumental variables (IVs), is instrumental in establishing causal relationships between exposures and clinical outcomes while controlling for confounders and mitigating reverse causation bias (Davies et al., 2018). Increasing evidence supports the utility of human genetic data related to gut microbial characteristics in clinical investigations, thereby positioning MR as a powerful tool to infer causal links between gut microbiota and ITP. In this study, we employed a two-step MR analysis and mediation analyses using summary statistics from the most extensive and current genome-wide association studies (GWAS) of the gut microbiota, plasma metabolites, and ITP. These analyses aimed to elucidate the intricate associations between these variables, providing valuable insights into their causal relationships.

## Methods

### Study design

**Figure 1:**
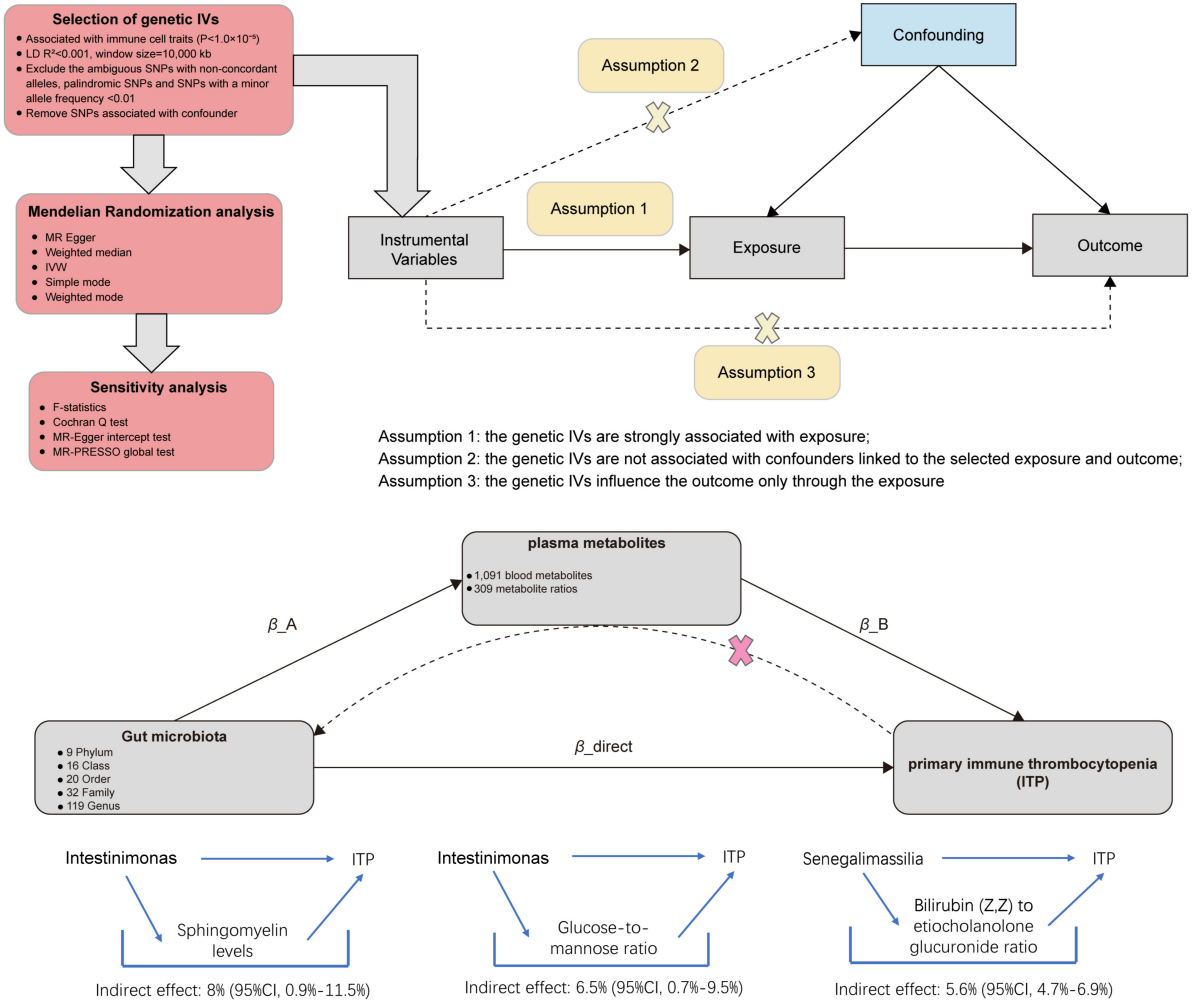
Diagrams illustrating associations examined in this study. The total effect between gut microbiota and primary immune thrombocytopenia (ITP). β_total is the total effect using genetically predicted gut microbiota as exposure and ITP as outcome. β_reverse is the total effect using genetically predicted ITP as exposure and gut microbiota as outcome. The total effect was decomposed into: (i) indirect effect using a two-step approach (where β_A is the total effect of gut microbiota on plasma metabolites, and β_B is the effect of plasma metabolites on ITP) and the product method (β_A × β_B) and (ii) direct effect (β_direct = β_total – β_A × β_B). Proportion mediated was the indirect effect divided by the total effect.

The study mainly conducted two steps of analyses as described in Figure 1: Step 1: the analysis of causal effects of 196 gut microbiota taxa on ITP; Step 2: analysis of candidate plasma metabolites as the mediation bridged the gut microbiota and ITP. In brief, the causal relationships between 1,400 plasma metabolites and ITP were analyzed. Then, the plasma metabolites positively associated with ITP were further screened for causal relationships with gut microbiota. Finally, the mediated effect of plasma metabolites was calculated. Mendelian randomization is based on three core assumptions: (1) the IVs are closely associated with the exposure factors. In our study, we ensured a strong correlation between genetic IVs and exposure factors (gut microbiota, plasma metabolites, or ITP) by removing linkage disequilibrium and strict correlation; (2) IVs are not associated with confounding factors. To ensure this, we used two sensitivity analysis methods (MR-PRESSO regression and MR Egger regression) to exclude the influence of confounding factors on the reliability of the results; (3) IVs do not affect the outcome directly, and it can only affect outcome via the exposure. All genetic instrumental variables were verified to have no association with the outcomes (Bowden and Holmes, 2019).

### Data sources

MiBioGen consortium designed and processed genome-wide genotypes and 16S fecal microbiome data from 18,340 individuals mainly from European background and the latest GWAS summary data included a total of 211 gut microbiota taxa (Kurilshikov et al., 2021). To enhancing the interpretability and scientific rigor of our findings, we deliberately excluded unknown microbial stains. So, a total of 196 microbiota taxa (119 genera, 32 families, 20 orders, 16 classes, and 9 phyla) were included in this study. The genetic data for metabolomics (comprising 1,091 metabolites and 309 metabolite ratios) from 8,299 unrelated European ancestry individuals were derived from the study by Yiheng Chen et al. and included in the GWAS Catalog (https://www.ebi.ac.uk/gwas/diagram) (Chen et al., 2023). The GWAS summary data of ITP from 456,348 European ancestry was acquired from the study by Jiang et al. (2021) and also included in the GWAS Catalog. Additional details are shown in Table 1. All GWAS data are from different consortia or organizations, and thus there is no overlapping sample.

**Table 1:** Characteristics of data in this study.

**Trait**		**Sample size**	**Population**	**Data source (PMID)**	**Description**
Gut microbiome	Phylum	18,340	European (16 cohorts, *N =* 13,266), Middle-Eastern (1 cohorts, *N =* 481), East Asian (1 cohort, *N =* 811), American Hispanic/Latin (1 cohort, *N =* 1,097), African American (1 cohort, *N =* 114), multi-ancestry (4 cohorts, *N =* 2,571)	MiBioGen consortium; www.mibiogen.org; (PMID:33462485)	The unknown texa of the samples were excluded
	Class				
	Order				
	Family				
	Genus				
Plamsa metabolites	Metabolites	8,299	European	GWAS Catalog; https://doi.org/10.1038/s41588-022-01270-1; (PMID:36635386)	Only European GWAS summary data: GCST90199621-90201020 were included
	Metabolite ratios				
Primary immune thrombocytopenia		456,348	European	GWAS Catalog; https://doi.org/10.1038/s41588-021-00954-4; (PMID:34737426)	

### Genetic instrumental variables (IVs) selection

For acquiring sufficient IVs, the displaying statistical significance of single nucleotide polymorphisms (SNPs) was set at *P*-value <1.0 × 10^−5^ (Malik et al., 2023). We extracted pertinent details including the chromosome (CHR), genomic location, effect allele (EA), other allele (OA), effect allele frequency (EAF) (if available), effect sizes (β), standard error (SE), *P-*value, and sample size (N). To ensure independence among the selected SNPs, a linkage disequilibrium (LD) threshold of r^2^ <0.001 was established, utilizing reference panel data from 1,000 Genomes Project Europeans samples (phase 3). This threshold facilitated the retention of independent SNPs with the lowest *P*-values (Bowden et al., 2015). Last, we calculated the explained variance (R^2^) and the F-statistic method was employed for SNP screening, calculated by dividing β by the square of the standard error, with a cut-off value set at 10 (Burgess et al., 2017). Subsequently, the identified SNPs were scrutinized using PhenoScanner V2 to identify potential confounding variables and confounders, including age, sex, race, and other diseases (Kamat et al., 2019).

### MR analysis

#### Primary analysis

We performed MR analysis in R software (version 4.4.3, http://www.r-project.org) with “Two-Sample MR” package (version 0.5.6) (Broadbent et al., 2020). In order to ascertain the causal effects of gut microbiota and plasma metabolites on ITP, we conducted two-sample MR analysis separately. The inverse variance weighted (IVW) approach served as the primary analysis method, while the Wald ratios test was utilized for features containing only one IV (Burgess et al., 2013). MR results were reported as odds ratios (ORs) along with their corresponding 95% confidence intervals (CIs). Statistical significance was determined when the *P*-value of the inverse variance weighted (IVW) method was <0.05. The exposure factors with no sufficient SNPs for harmonization were excluded.

#### Reverse causality analysis

To assess reverse directional causation effects between gut microbiota and primary immune thrombocytopenia (ITP), we considered ITP as the “exposure” and gut microbiota associated with ITP as the “outcome.” SNPs significantly associated with ITP (*P* < 1 × 10^−5^) were selected as instrumental variables (IVs).

#### Sensitivity analysis

The “MR_PRESSO” package was employed for multiplicity testing (Ong and MacGregor, 2019). Cochran's Q test was conducted to assess the heterogeneity of each SNP (Cohen et al., 2015). Leave-one-out analysis was performed to assess the influence of each SNP on the overall results (Burgess and Thompson, 2017). Furthermore, MR-PRESSO regression and MR-Egger regression were utilized to examine potential horizontal pleiotropy effects (Verbanck et al., 2018).

#### Mediation analysis

In the two-sample analysis, gut microbiota and plasma metabolites showing significant causal effects on ITP were selected for mediation analysis. We investigated whether gut microbiota had a causal effect on plasma metabolites, which in turn had causal effects on ITP. The percentage mediated by the mediating effect was calculated by dividing the indirect effect by the total effect. 95% confidence intervals were computed using the “RMediation” package. Results with *P* < 0.05 and mediation percentages between 0% and 100% were considered statistically meaningful (Tofighi and MacKinnon, 2011).

## Results

### Causal effects of gut microbiota on ITP

**Figure 2:**
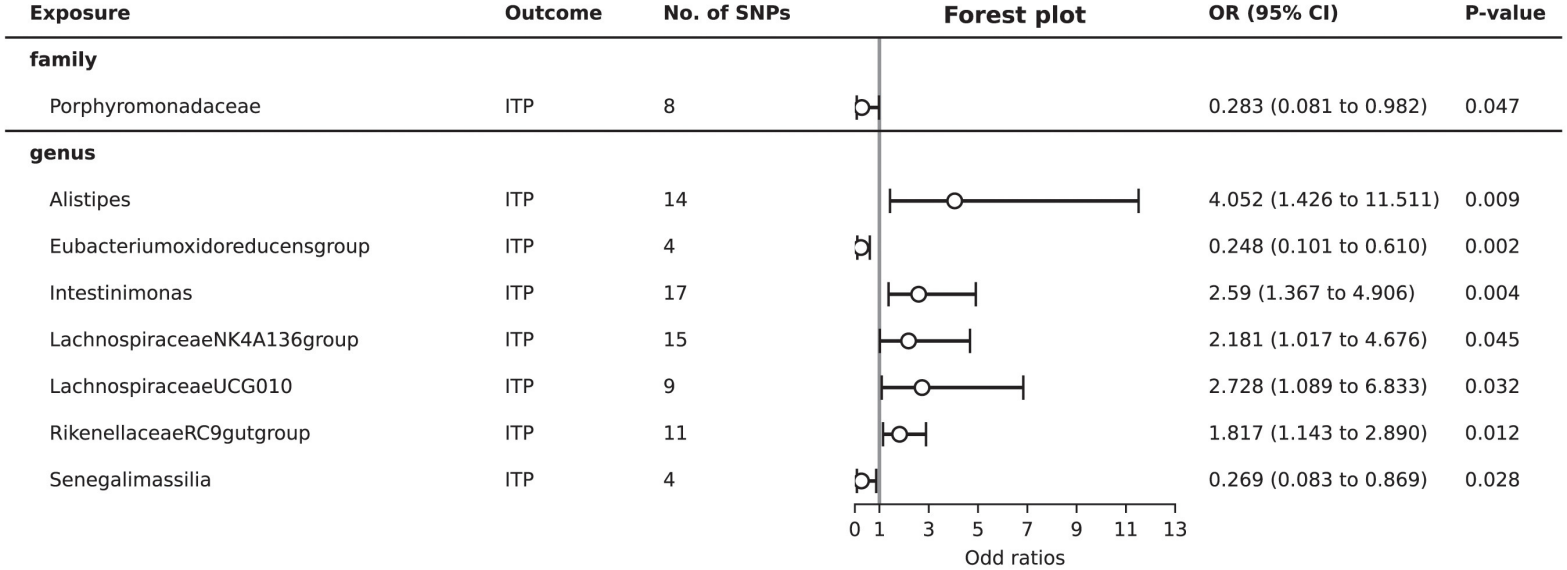
Forest plot to visualize the significant causal effects of gut microbiota on ITP.

Total of eight gut microbiota (including one family, seven genera) were associated with ITP (Supplementary Table S3, Figure 2). Detailed 2,182 SNPs information for included microbiota is shown in Supplementary Table S1.

As shown in Figure 2, MR analysis suggested that genetic prediction of five gut microbiota (genus *Alistipes*, genus *Intestinimonas*, genus *Lachnospiraceae NK4A136 group*, genus *Lachnospiraceae UCG010*, and genus *Rikenellaceae RC9 gut group*) was associated with an increased risk of ITP. The genus *Alistipes* (OR = 4.052, 95%CI = 1.426–11.511, *P* = 0.009), genus *Intestinimonas* (OR = 2.590, 95%CI = 1.367–4.906, *P* = 0.004), genus *Lachnospiraceae NK4A136 group* (OR = 2.181, 95%CI = 1.017–4.676, *P* = 0.045), genus *Lachnospiraceae UCG010* (OR = 2.728, 95%CI = 1.089–6.833, *P* = 0.032), and genus *Rikenellaceae RC9 gut group* (OR = 1.817, 95%CI = 1.143–2.890, *P* = 0.012) significantly increased the risk of ITP.

Genetic prediction of three gut microbiota (family *Porphyromonadaceae*, genus *Eubacterium oxidoreducens group*, and genus *Senegalimassilia*) was associated with a decreased risk of ITP. The family *Porphyromonadaceae* (OR = 0.283, 95%CI = 0.081–0.982, *P* = 0.047), genus *Eubacterium oxidoreducens group* (OR = 0.248, 95%CI = 0.101–0.610, *P* = 0.002), and genus *Senegalimassilia* (OR = 0.269, 95%CI = 0.083–0.869, *P* = 0.028) significantly decreased the risk of ITP.

According to the MR-Egger regression intercept approach and MRPRESSO analysis, horizontal pleiotropy was observed in the family *Oxalobacteraceae*, genus *Eggerthella*, genus *ErysipelotrichaceaeUCG003*, genus *Oscillibacter*, genus *Peptococcus*, genus *Anaerofilum*, and genus *Ruminiclostridium5* in the MR study. The Cochran's Q tests indicated heterogeneity in the MR results for the family *Actinomycetaceae*, genus *Actinomyces*, genus *Anaerofilum*, genus *Butyricimonas*, genus *Eubacterium brachy group*, genus *Eubacterium rectale group*, genus *Eubacterium ventriosum group*, genus *Hungatella*, genus *Olsenella*, genus *Ruminiclostridium5*, and genus *Veillonella*. These findings do not affect the identification of the eight gut microbiota with a causal relationship with ITP in the MR analysis, ensuring the reliability of the results (Supplementary Table S2).

### Reverse causal effects of ITP on gut microbiota

As shown in Supplementary Table S6, ITP has causal effect on 7 of 8 gut microbiota [family *Porphyromonadaceae* (OR = 1.024, 95%CI = 1.013–1.036, *P* < 0.001), genus *Alistipes* (OR = 1.029, 95%CI = 1.021–1.038, *P* < 0.001), genus *Eubacterium oxidoreducens group* (OR = 0.964, 95%CI = 0.948–0.980, *P* < 0.001), genus *LachnospiraceaeNK4A136group* (OR = 1.018, 95%CI = 1.009–1.026, *P* < 0.001), genus *LachnospiraceaeUCG010* (OR = 0.986, 95%CI = 0.976–0.995, *P* = 0.003), genus *RikenellaceaeRC9gutgroup* (OR = 0.938, 95%CI = 0.918–0.959, *P* < 0.001), and genus *Senegalimassilia* (OR = 0.964, 95%CI = 0.943–0.986, *P* = 0.001)] identified with causal effect on ITP. Detailed 335 SNPs information for reverse causal analyses is shown in Supplementary Table S4.

In the MR-Egger regression intercept and MRPRESSO analysis, horizontal pleiotropy was detected for the associations between ITP and genus *Alistipes*, genus *Eubacterium oxidoreducens group*, and family *Porphyromonadaceae*. Cochran's Q tests indicated that the causal relationships between ITP and family *Porphyromonadaceae*, genus *Senegalimassilia*, and genus *Senegalimassilia* were influenced by heterogeneity. Based on these MR results, we identified reverse causal confounding between ITP and genus *Lachnospiraceae NK4A136 group*, genus *Lachnospiraceae UCG010*, and genus *Rikenellaceae RC9 gut group*, leading to their exclusion from subsequent mediation analysis (Supplementary Table S5).

### Causal effects of plasma metabolites on ITP

**Figure 3:**
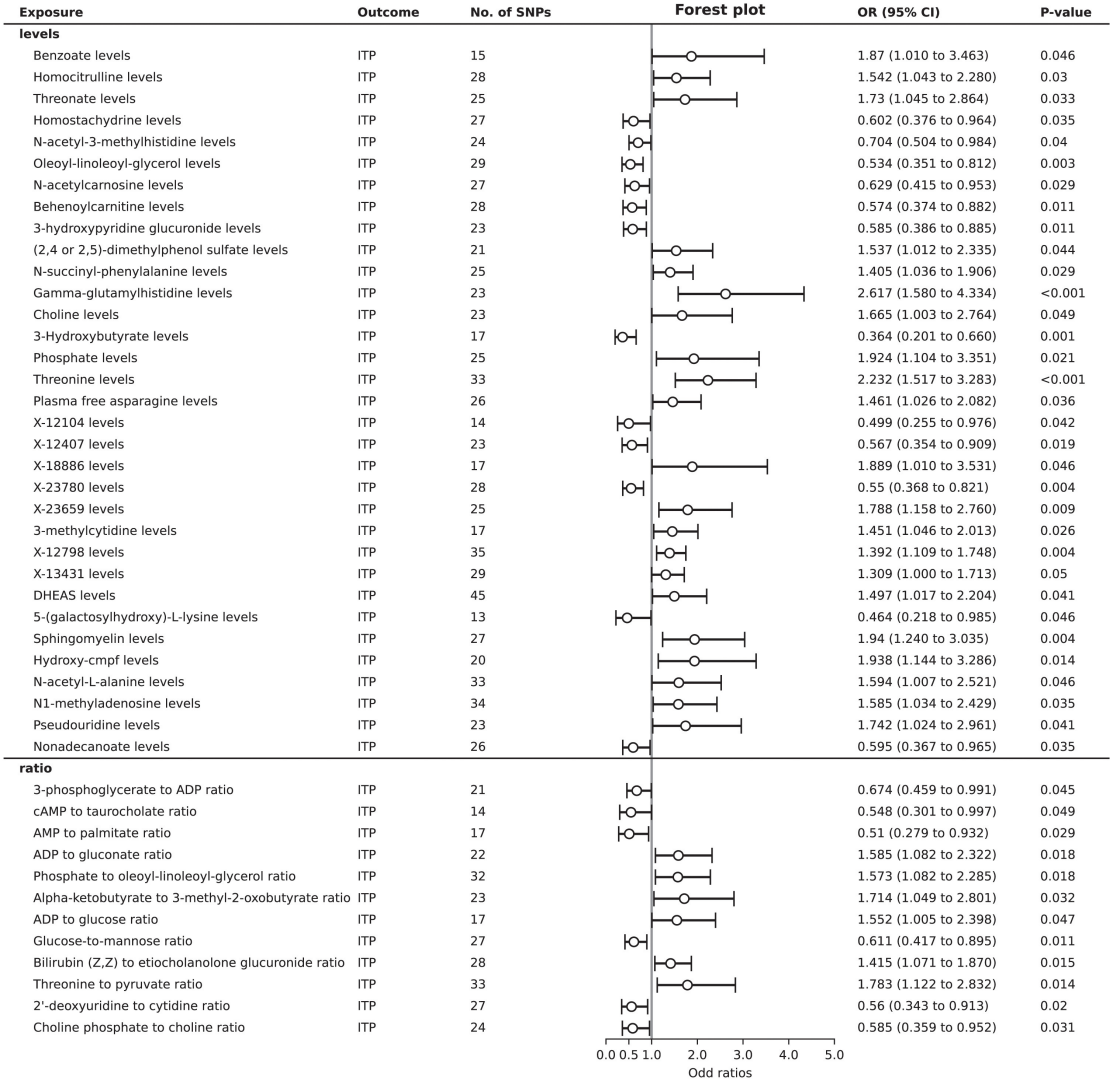
Forest plot to visualize the significantly causal effects of plasma metabolites on ITP.

A total of 45 plasma metabolites were found to be associated with ITP. Among the levels of plasma metabolites, 21 showed a positive causal relationship with ITP, while 12 exhibited a negative causal relationship. Regarding the ratios of plasma metabolites, elevated levels of 6 ratios were identified as risk factors for ITP, whereas elevated levels of another 6 ratios were identified as protective factors (Figure 3). Detailed 1,124 SNPs information for MR analysis of 45 plasma metabolites on ITP is shown in Supplementary Table S7.

In the sensitivity analysis results for 45 plasma metabolites, Cochran's Q-test showed no evidence of heterogeneity in the causal relationship between these SNPs. The results of the MR-Egger regression intercept indicated potential horizontal pleiotropy for threonate levels, behenoylcarnitine (C22) levels, X-23780 levels, the phosphate to oleoyl-linoleoyl-glycerol (18:1 to 18:2) (Bussel et al., 2023) ratio, nonadecanoate (19:0) levels, and the 2′-deoxyuridine to cytidine ratio. Conversely, the MR-PRESSO test did not identify significant pleiotropy. To maximize the discovery of plasma metabolites potentially mediating the causal link between gut microbiota and ITP, we included the levels or ratios of these 45 plasma metabolites in the subsequent analysis (Supplementary Table S8).

### Causal effects of gut microbiota on plasma metabolites associated to ITP

**Figure 4:**
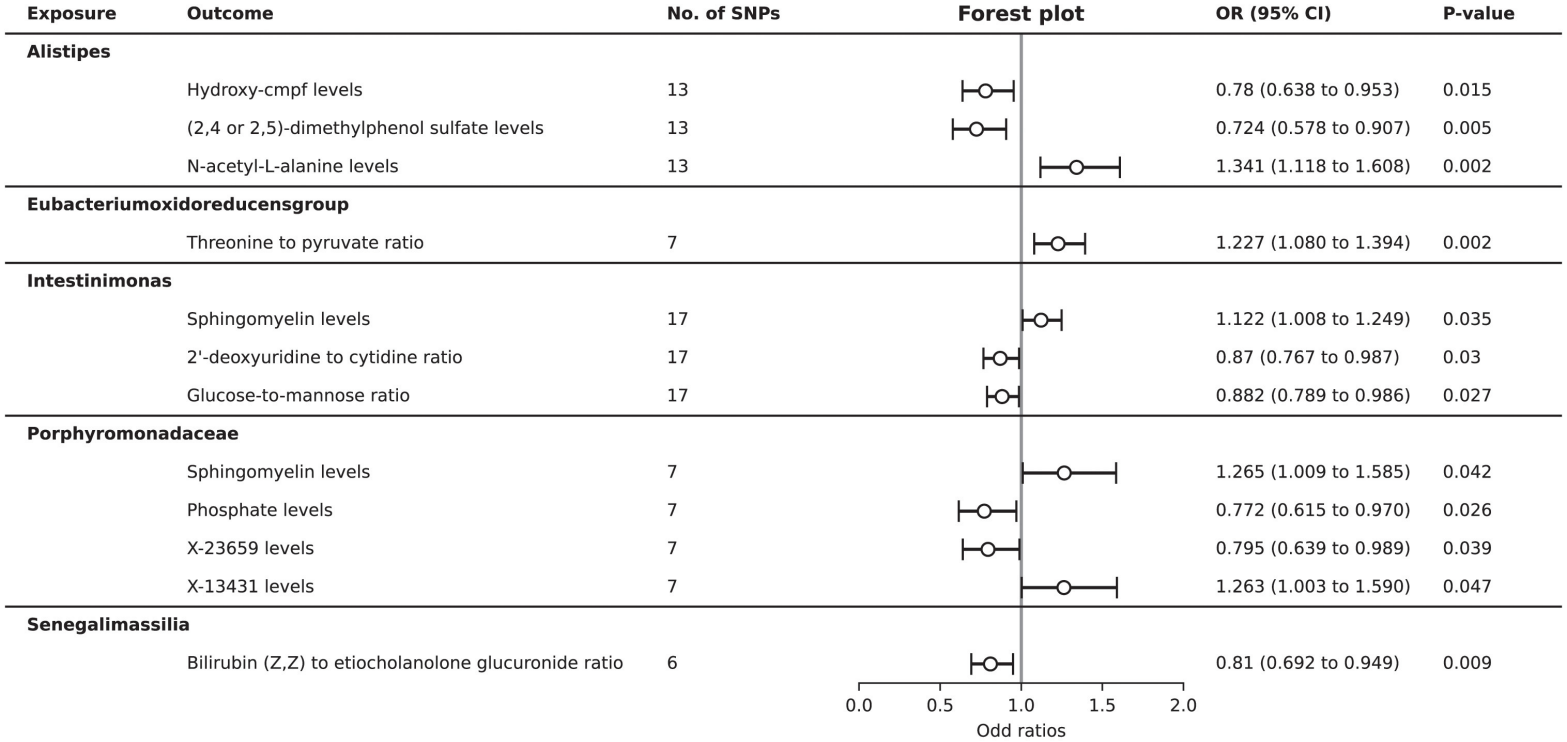
Forest plot to visualize the causal effects of gut microbiota on plasma metabolites associated with ITP.

After harmonization, there were 2,836 SNPs were filtered for further MR analysis (Supplementary Table S10). A total of 12 causal relationships were identified between the 8 gut microbiota and 45 plasma metabolites associated with ITP (Supplementary Table S12). Specifically, the genus *Alistipes* was found to have a protective effect on the plasma levels of hydroxy-cmpf (OR = 0.780, 95% CI = 0.638–0.953, *P* = 0.015) and (2,4 or 2,5)-dimethylphenol sulfate (OR = 0.724, 95% CI = 0.578–0.907, *P* = 0.005), while it was a risk factor for N-acetyl-L-alanine levels (OR = 1.341, 95% CI = 1.118–1.608, *P* = 0.002). The genus *Eubacterium oxidoreducens group* was identified as a risk factor for the ratio of threonine to pyruvate (OR = 1.227, 95% CI = 1.080–1.394, *P* = 0.002). The genus *Intestinimonas* was a risk factor for sphingomyelin levels (OR = 1.122, 95% CI = 1.008–1.249, *P* = 0.035) and a protective factor for the 2′-deoxyuridine to cytidine ratio (OR = 0.870, 95% CI = 0.767–0.987, *P* = 0.030) and the glucose-to-mannose ratio (OR = 0.882, 95% CI = 0.789-0.986, *P* = 0.027). The family *Porphyromonadaceae* may promote the levels of sphingomyelin (OR = 1.265, 95% CI = 1.009–1.585, *P* = 0.042) and X-13431 (OR = 1.263, 95% CI = 1.003–1.590, *P* = 0.047), while inhibiting the levels of phosphate (OR = 0.772, 95% CI = 0.615–0.970, *P* = 0.026) and X-23659 (OR = 0.795, 95% CI = 0.639–0.989, *P* = 0.039). The genus *Senegalimassilia* was negatively associated with the ratio of bilirubin (Z,Z) to etiocholanolone glucuronide (OR = 0.810, 95% CI = 0.692–0.949, *P* = 0.009) (Figure 4).

Although seven pairs of causal relationships between the included gut microbiota and plasma metabolites did not pass the sensitivity analysis, the MR-Egger regression intercept approach and the MRPRESSO analysis indicated that heterogeneity and genetic pleiotropy did not bias the significant results (Supplementary Table S11).

### Mediation analysis of gut microbiota on ITP via plasma metabolites

Based on the above analysis, gut microbiota and plasma metabolites both have causal effects on ITP. It appears that plasma metabolites mediate the pathway between gut microbiota and ITP. Using the delta method, we identified Sphingomyelin levels (β = 0.076, 95% CI = 0.003–0.183, *P* = 0.047) and the Glucose-to-mannose ratio (β = 0.062, 95% CI = 0.002–0.151, *P* = 0.039) as mediators in the effect of genus *Intestinimonas* on ITP. Additionally, the Bilirubin (Z,Z) to etiocholanolone glucuronide ratio (β = −0.073, 95% CI = −0.171–−0.007, *P* = 0.043) was identified as a mediator in the effect of genus *Senegalimassilia* on ITP. In terms of mediation proportion, Sphingomyelin levels and the Glucose-to-mannose ratio accounted for 8% (95% CI = 0.9%−11.5%) and 6.5% (95% CI = 0.7%−9.5%) of the effect of genus *Intestinimonas* on ITP, respectively. The Bilirubin (Z,Z) to etiocholanolone glucuronide ratio accounted for 5.6% (95% CI = 4.7%−6.9%) of the effect of genus *Senegalimassilia* on ITP (Table 2).

**Table 2:** The mediation effect of gut microbiota on primary immune thrombocytopenia (ITP) via plama metabolites.

**Exposure.Taxa**	**Mediator**	**Total effect**	**Direct effect A**	**Direct effect B**	**Mediation effect**		**Mediated proportion (%) (95% CI)**
		β **(95% CI)**	β **(95% CI)**	β **(95% CI)**	β **(95% CI)**	***P*** **-value**	
Intestinimonas	Sphingomyelin levels	0.952 (0.312 to 1.591)	0.115 (0.008 to 0.223)	0.663 (0.215 to 1.110)	0.076 (0.003 to 0.183)	0.047	8 (0.9 to 11.5)
Intestinimonas	Glucose-to-mannose ratio	0.952 (0.312 to 1.591)	−0.125 (−0.237 to −0.014)	−0.492 (−0.874 to −0.111)	0.062 (0.002 to 0.151)	0.039	6.5 (0.7 to 9.5)
Senegalimassilia	Bilirubin (Z,Z) to etiocholanolone glucuronide ratio	−1.313 (−2.485 to −0.140)	−0.211 (−0.369 to −0.053)	0.347 (0.068 to 0.626)	−0.073 (−0.171 to −0.007)	0.043	5.6 (4.7 to 6.9)

Total ellect indicates the elect of intestinal microbiota on primary immune thrombocytopenia (ITP), “direct effect A” indicates the effect of intestinal microbiota on plama metabolites, “direct effect B” indicates the effect of plama metabolites on primary immune thrombocytopenia (ITP) 'mediation effect' indicates the effect of intestinal microbiota on primary immune thrombocytopenia (ITP) through plama metabolites. Total effect, direct effect A and direct effect B were derived by IVW; mediation effect was derived by using the delta method. All statistical tests were two-sided. *P* < 0.05 was considered significant.

## Discussion

This study is the first to identify plasma mediators influenced by the gut microbiota in immune thrombocytopenia (ITP) from a big data perspective. We conducted a large-scale two-sample Mendelian randomization analysis using summary data from the MiBioGen consortium GWAS meta-analysis, alongside plasma metabolites and ITP data summarized from relevant literature, to identify plasma metabolites mediating the causal relationship between gut microbiota and ITP. Our Mendelian randomization analysis revealed a unidirectional causal relationship between five gut microbiota taxa (family *Porphyromonadaceae*, genus *Alistipes*, genus *Eubacterium oxidoreducens group*, genus *Intestinimonas*, and genus *Senegalimassilia*) and ITP. Among the 45 plasma metabolites with a causal relationship with ITP, sphingomyelin levels, glucose-to-mannose ratio, and bilirubin (Z,Z) to etiocholanolone glucuronide ratio mediated the effects of genus *Intestinimonas* and genus *Senegalimassilia* on ITP.

Host-microbiota interactions are crucial for host physiology and disease phenotypes, with dysbiosis potentially promoting disease development through alterations in pathogens and their associated metabolites (Elsouri et al., 2021; Opoku et al., 2022). Current evidence suggests bidirectional communication between gut microbiota composition and function and the occurrence and progression of Immune Thrombocytopenia (ITP) (Li et al., 2023). As an example, one group reported antibodies against platelets were identified on one female patient's platelets who developed thrombocytopenia after two fecal microbial transplantations (Malnick et al., 2015). In our study, among identified 8 gut microbiota taxa causally linked to ITP, genus *Lachnospiraceae NK4A136 group*, genus *Lachnospiraceae UCG010*, and genus *Rikenellaceae RC9* gut group, exhibited bidirectional causal relationships with ITP. Among the microbiota promoting ITP, we identified genus *Alistipes* within the phylum *Bacteroidetes*, consistent with previous reports of a decreased *Firmicutes/Bacteroidetes* ratio in ITP (Yu et al., 2022). Additionally, the causal relationship between genus *Intestinimonas* within the family *Lachnospiraceae* and ITP parallels findings by Dongmei Guo, which demonstrated a causal link between *Lachnospiraceae* and ITP based on different outcome cohorts (Guo et al., 2023). In terms of protective factors against ITP development, family *Porphyromonadaceae* and genus *Eubacterium oxidoreducens group* were identified as significant protective factors. Although their roles in ITP have not been previously reported, the protective effects of specific microbiota within the family *Porphyromonadaceae* against autoimmune diseases are well-established (Mao et al., 2023). Moreover, some discrepancies between our results and previous Mendelian randomization studies on the causal relationship between gut microbiota and ITP may be attributed to the heterogeneity in ITP etiology and differences in the outcome cohort data utilized (Guo et al., 2023; Jiang et al., 2024).

Metabolites are intermediate or end products of metabolic reactions, with plasma metabolite levels influenced by various factors such as gut microbiota and medications (Lavelle and Sokol, 2020). Changes in plasma metabolites can affect disease outcomes and serve as potential therapeutic targets (Yoon et al., 2021). Using GC-MS technology, the potential associations between ITP and plasma metabolites have been gradually uncovered (Zhang et al., 2022); however, systematic studies on their causal relationships are lacking. Through Mendelian randomization analysis, this study newly identified 45 plasma metabolites with potential causal links to ITP. Fujii et al. (2021) discovered that defects in sphingomyelin synthase 1 lead to thrombocytopenia, which aligns with our finding that Sphingomyelin level is a protective factor for ITP. Importantly, we identified that genus *Intestinimonas* promotes plasma Sphingomyelin levels, thereby protecting against ITP from the perspective of gut microbiota. Additionally, an increase in mannose in platelets has been observed in ITP patients (Ramírez-López et al., 2021); our study validated that the Glucose-to-mannose ratio acts as a negative regulatory mediator in the effect of genus *Intestinimonas* on ITP. In summary, the reported associations between metabolites and ITP are consistent with the causal relationships identified in our study.

Investigations into the gut microbiota and metabolites indicate that their changes are closely related to the clinical characteristics of ITP (Wang et al., 2023). For example, it has been reported that among ITP patients receiving treatment, the increased abundance of *Pseudomonas* in the gut suggests a mechanism for good prognosis (Rui et al., 2023). Enzymes produced by *Pseudomonas*, such as alkaline protease and elastase, have been shown to play a role in inhibiting the activity of neutrophils and natural killer cells, while also inhibiting lymphocyte proliferation by proteolytic hydrolysis of IL-2, ultimately improving ITP (Theander et al., 1988). Likely, our found plasma metabolites causally associated to a certain of gut microbiota are potential biomarkers reflecting the severity of ITP during disease progression. On the other hand, there seems to be a bidirectional relationship between the composition of the microbiota and treatment in ITP patients. For example, the genus *Lachnospiraceae* is clearly present in the gut microbiota of ITP and RA patients (Wu et al., 2016). In recent years, probiotics and prebiotics have been recommended for the treatment of various diseases, including autoimmune diseases. Thereby, our explored three gut microbiota-plasma metabolites-ITP relationship are ideal candidate probiotics and prebiotics hopefully utilized in the treatment of ITP.

Our study exploring the relationships between gut microbiota, plasma metabolites, and ITP encountered several limitations. Firstly, the generalizability of our findings is constrained by the predominantly European ancestry of our sample, which may not accurately reflect the genetic and lifestyle diversity influencing gut microbiota in different populations. Additionally, the use of 16S *rRNA* gene sequencing provides taxonomic insights only up to the genus level, lacking the depth of species-level analysis that metagenomic sequencing could offer. The associations observed between certain microbiota and ITP risk are preliminary, and given the complexity of interactions within the gut microbiome and with ITP, these findings should be interpreted with caution. Moreover, our approach based on Mendelian randomization (MR) may not fully capture the intricate, potentially non-linear relationships between gut microbiota and ITP. It's necessary to perform prospective cohort studies to help further validate these causal associations. The current limitations of available GWAS datasets for ITP also hinder our ability to conduct reliable replication studies using diverse GWAS data, which is crucial for establishing definitive causal relationships. The relatively small sample size of existing ITP GWAS datasets reduces the power to detect true causal effects of certain exposures, potentially increasing the risk of false-negative results. Therefore, future research requires larger GWAS datasets to validate our findings and further elucidate the complex role of gut microbiota in the pathogenesis of ITP. Lastly, the hypothesis-driven nature of MR emphasizes the detection of biologically plausible causal relationships. Thus, our exploratory work prioritizes biologically meaningful associations that should be further investigated in larger datasets in future studies.

To our knowledge, this is the first comprehensive study to evaluate the causal relationships between gut microbiota, plasma metabolites, and ITP. We have identified a significant role for gut microbiota and serum metabolites in the pathogenesis and progression of ITP in the host. These findings underscore the necessity of further exploring the mechanisms underlying the interactions between gut microbiota and ITP and deeper mechanisms based on individual-level data and experimental studies will be further explored to gain a more nuanced understanding. Moreover, our results provide novel insights that may inform the development of microbiota-based therapies and plasma metabolite-targeted interventions for ITP.

## Data Availability

The original contributions presented in the study are included in the article/Supplementary material, further inquiries can be directed to the corresponding authors.
